# Ultratrace Detection of Histamine Using a Molecularly-Imprinted Polymer-Based Voltammetric Sensor

**DOI:** 10.3390/s17030645

**Published:** 2017-03-21

**Authors:** Maedeh Akhoundian, Axel Rüter, Sudhirkumar Shinde

**Affiliations:** Department of Biomedical Sciences, Faculty of Health and Society, Malmö University, Malmö SE-20506, Sweden; m.akhoundian@yahoo.com (M.A.); axel.ruter@fkem1.lu.se (A.R.)

**Keywords:** histamine imprinted polymers, Ultratraces, sensor

## Abstract

Rapid and cost-effective analysis of histamine, in food, environmental, and diagnostics research has been of interest recently. However, for certain applications, the already-existing biological receptor-based sensing methods have usage limits in terms of stability and costs. As a result, robust and cost-effective imprinted polymeric receptors can be the best alternative. In the present work, molecularly-imprinted polymers (MIPs) for histamine were synthesized using methacrylic acid in chloroform and acetonitrile as two different porogens. The binding affinity of the MIPs with histamine was evaluated in aqueous media. MIPs synthesized in chloroform displayed better imprinting properties for histamine. We demonstrate here histamine MIPs incorporated into a carbon paste (CP) electrode as a MIP-CP electrode sensor platforms for detection of histamine. This simple sensor format allows accurate determination of histamine in the sub-nanomolar range using an electrochemical method. The sensor exhibited two distinct linear response ranges of 1 × 10^−10^–7 × 10^−9^ M and 7 × 10^−9^–4 × 10^−7^ M. The detection limit of the sensor was calculated equal to 7.4 × 10^−11^ M. The specificity of the proposed electrode for histamine is demonstrated by using the analogous molecules and other neurotransmitters such as serotonin, dopamine, etc. The MIP sensor was investigated with success on spiked serum samples. The easy preparation, simple procedure, and low production cost make the MIP sensor attractive for selective and sensitive detection of analytes, even in less-equipped laboratories with minimal training.

## 1. Introduction

Histamine (β-imidazolylethylamine) is a biogenic amine. It is an important mediator involved in various physiological and pathological processes, including neurotransmission and numerous brain functions, secretion of some hormones, regulation of gastrointestinal, circulatory functions, and inflammatory reactions [1]. However, a high level of histamine in the human body causes an allergy-like syndrome called histamine intolerance [2] or histamine poisoning (toxic level 50 mg per 100 g of product) [3,4]. The source of elevated histamine level is due to fermented foods, such as fish, cheese, sauerkraut, beer, wine, processed meat [2], and spoilage of the foodstuff, in particular seafood, due to uncontrolled microbial growth [5]. Therefore, monitoring of histamine is a critical task for the food industry and food safety.

A number of methods for the determination of histamine have been reported including thin-layer chromatography [6,7], gas chromatography [8,9], capillary zone electrophoresis [10], and high-performance liquid chromatography [11,12,13,14], as well as fluorimetric [15] and colorimetric assays [16]. These methods require extensive sample processing such as pretreatment of samples. In addition, it involves qualified analysts. These tedious and time-consuming methods result in expensive and slow sample throughput. In this regard, enzyme-based methods [e.g., enzyme-linked immunosorbent assay (ELISA)] [17,18] offer rapid means of detection, but necessitate the use of unstable enzymes, expensive test kits, and tend to overestimate histamine [19].

Different types of electrochemical sensors with chemical modifications [20,21,22] or with immobilized amine oxidases and dehydrogenases [23,24,25,26,27,28] have been described in several reports of histamine determination.

The development of stable histamine receptor with a capacity to detect low histamine concentrations (nM range) is an urgent need in the biomedical and diagnostics research [29,30,31,32,33,34]. In this direction, MIP-based sensors have attracted much interest due to easy preparation, good stability, and robustness. In the literature, histamine-imprinted polymers and MIP-based sensors have already been reported for histamine recognition [35,36,37] in surface enhanced Raman spectroscopy (SER) [38], thermal [39], quartz crystal microbalance (QCM) [40], amperometric [41], and impedimetric [34,42] sensors.

In the current work, a histamine MIP has been developed and used for fabrication of voltammetric sensor. The developed sensor has been successfully applied for histamine determination in serum samples. The developed methodology offers advantages such as simplicity, precision, short analysis time, low cost of analysis and instrumentation, with comparable selectivity and sensitivity with advanced instruments.

## 2. Materials and Methods

Dopamine hydrochloride, histamine, and serotonin hydrochloride was received from Sigma, (Steinheim, Germany). H-His-OH (99%) was received from Bachem Biochemica GmBH (Heidelberg, Germany) and Boc-His-OH was received from Calbiochem-Novabiochem AG (Läufelfingen, Switzerland). Methacrylic acid (MAA) and ethylene glycol dimethacrylate (EGDMA) were purchased from Sigma-Aldrich Chemie GmbH (Taufkirchen, Germany). EGDMA was washed consecutively with 10% NaOH, water, and brine and then dried over MgSO_4_, and filtered prior to distillation under reduced pressure. MAA was also distilled under reduced pressure. The initiator azo-N,N’-bisdivaleronitrile (ABDV) was purchased from Wako Chemicals and used without further purification. Chloroform (CHCl_3_), extra dry, and acetonitrile (MeCN), extra dry, were received from Acros Organics (Geel, Belgium). The porogens were kept under nitrogen atmosphere over molecular sieves and were used without further purification. Graphite flake powder (325 mesh) was received from Alfa Aesar and carbon (mesoporous nanopowder, ˂500 nm particle size) was received from Aldrich (Steinheim, Germany). Paraffin oil was received from Kebo AB (Stockholm, Sweden).

### 2.1. Polymer Synthesis

Imprinted polymers were prepared using bulk polymerization (Table 1) in the following manner: Template histamine (0.24 mmol), functional monomer MAA (1.2 mmol), and EDGMA (6 mmol) were dissolved in 1.5 mL dry chloroform (MIP1) and 1.5 mL dry acetonitrile (MIP2). The initiator ABDV (1% *w/w* of total monomers) was added to the solution which was transferred to screw-capped glass vials, cooled to 0 °C, and purged with a flow of dry nitrogen for 10 min. The glass vials were sealed with silicone tape while still under cooling and the polymerization was initiated by placing the vials at 50 °C for 48 h. The polymers were lightly crushed and the template was extracted with methanol: acetic acid/90:10 for 24 h. This was followed by further lightly crushing the particles without fractionation to evaluate their binding properties. Corresponding non-imprinted polymers (NIP1/NIP2) were prepared in the same manner described above, but with the omission of the template molecule from the pre-polymerization solution.

### 2.2. Binding Analysis

Increasing amount of histamine (0 to 1 mM) 1 mL solution was suspended in the 10 mg MIP and NIP particles. Rebinding tests of the polymeric materials were all performed in a 50 mM PBS solution (pH 7.4) with an equilibration time of 4 h followed by analysis with UV-VIS spectroscopy measuring absorbance at 208 nm. Measurements were performed on a BIOTEK POW-ERWAVE XS plate reader in a 96-well quartz plate.

### 2.3. Preparation of the Sensors

In order to fabricate the sensor, 11 mg of nano-carbon (NC) and 0.2 g of graphite was dispersed in 2 mL of dimethylformamide (DMF) and sonicated for 30 min, and allowed to dry in an oven at 80 °C overnight. This mixture was then added to the 11 mg of MIP1/NIP1 and homogenized in a mortar. Subsequently, 57.6 mg of paraffin oil was added to the mixture to form a homogeneous paste. This paste was used to fill a hole (4.00 mm in diameter, 3 mm in depth) at the end of the electrode body. The excess material from the surface of the electrode was removed by polishing on a paper sheet, thus devising the MIP-CP electrode. For the normal mixing electrode, all of the steps were repeated except the dispersion and sonication steps otherwise described.

### 2.4. General Method for Electrochemical Measurements

Electrochemical data was obtained with a system using an Ivium-stat potentiostat/galvanostat. The measurements were performed in a three-electrode system: working electrode (MIP/NIP-based CP electrodes), a counter electrode (platinum), and a reference electrode (Ag/AgCl). Electrochemical measurement of histamine concentration was performed according to the following procedure: The solution containing 20 mL of potassium hexacyanoferrate (0.1 M K_3_[Fe(CN)_6_]) and potassium chloride (0.1 M KCl) was added to the cell as a blank. Before each determination, histamine stock solution was prepared in PBS (0.1 M, pH = 7), spiked to the blank solution and stirred for 15 s. The MIP-CP electrode was immediately placed into the electrochemical cell and the cyclic voltammetry technique was applied using scan rate = 50 mV/s, Estep = 10 mV, and equilibration time = 5 s. The modified electrode was rinsed with milliQ water and polished on the paper sheet after each measurement.

### 2.5. The Measurement of Histamine in Real Samples

In order to determine the histamine in real sample, a certain amount of histamine stock solution was spiked in to the serum sample and it was diluted with milliQ water in a 1:20 ratio. The prepared sensor was then immersed in to the spiked serum sample and solution was stirred for 15 s. Cyclic voltammetry responses were recorded immediately after equilibration time of the program, which was 5 s.

## 3. Results

### 3.1. Molecularly- Imprinted Polymers for Histamine

#### 3.1.1. Polymer Synthesis

The preparation of histamine MIPs was done following established procedure with slight modifications (Table 1) starting from prepolymerization mixtures containing histamine as the template, methacrylic acid (MAA) as functional monomers, and ethylenglycol dimethacrylate (EGDMA) as a crosslinking monomer. In order to study the effect of porogen on histamine imprinting, we chose CHCl_3_ and MeCN over highly-hygroscopic polar dimethyl sulfoxide (DMSO) among mostly used porogens reported earlier. After free radical polymerization, the resulting polymers were freed from the template by washing with acidic methanol, leaving binding sites complementary to a relatively narrow group of histamine.

#### 3.1.2. Optical Batch Rebinding Experiment

**Figure 1:**
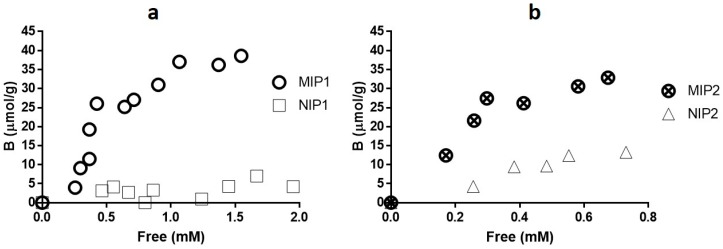
Rebinding isotherm of histamine (**a**) MIP1, NIP1; (**b**) MIP2, NIP2 in 50 mM PBS buffer (pH 7.4). MIP1 and MIP2 (corresponding NIPs) were prepared in CHCl_3_ and MeCN, respectively.

At this stage, we were interested in measuring histamine in biological fluids at neutral pH and aqueous environment. The functional monomer MAA [43] (the pKa 6.5), which was used by Bongaers et al. [42], Horemans et al. [40], and Trikka et al. [35], is a deprotonated form (COO^−^) at neutral pH, which binds to histamine. The MIP synthesized from MAA is suitable for rebinding of histamine at pH 7.4 [34,35]. Therefore, we performed rebinding experiments for synthesized MIPs at pH 7.4 using PBS buffer followed by analysis with UV spectrophotometer. For the rebinding experiment, 10 mg of MIP and NIP powder were added to 1 mL of histamine between 0 to 1 mM concentration ranges. The resulting suspensions were shaken for 4 h at room temperature. The supernatant was collected after centrifugation and the free concentration of histamine was determined by UV-VIS spectroscopy. Hereby, the amount of bound histamine per gram of MIP or NIP was calculated and the binding isotherms were constructed. The binding isotherms for the MIP and NIP were prepared in CHCl_3_ and MeCN are demonstrated in Figure 1a,b. The polymer prepared in CHCl_3_ displayed better imprinting performance compared to those in MeCN. This is due to the difference in polarity (chloroform is less polar than acetonitrile) a large concentration of prepolymer complex would be expected using chloroform as a porogen [44]. Further, Trikka et al. [35] has confirmed by ^1^H NMR spectroscopy that interaction between histamine and MAA are mainly hydrogen bonding when chloroform is used as a solvent. The best performing MIP and NIP prepared in CHCl_3_ was further used for MIP-modified carbon paste (CP) electrodes.

### 3.2. MIP-CP Electrode

#### 3.2.1. Fabrication of MIP-CP Electrode

**Figure 2:**
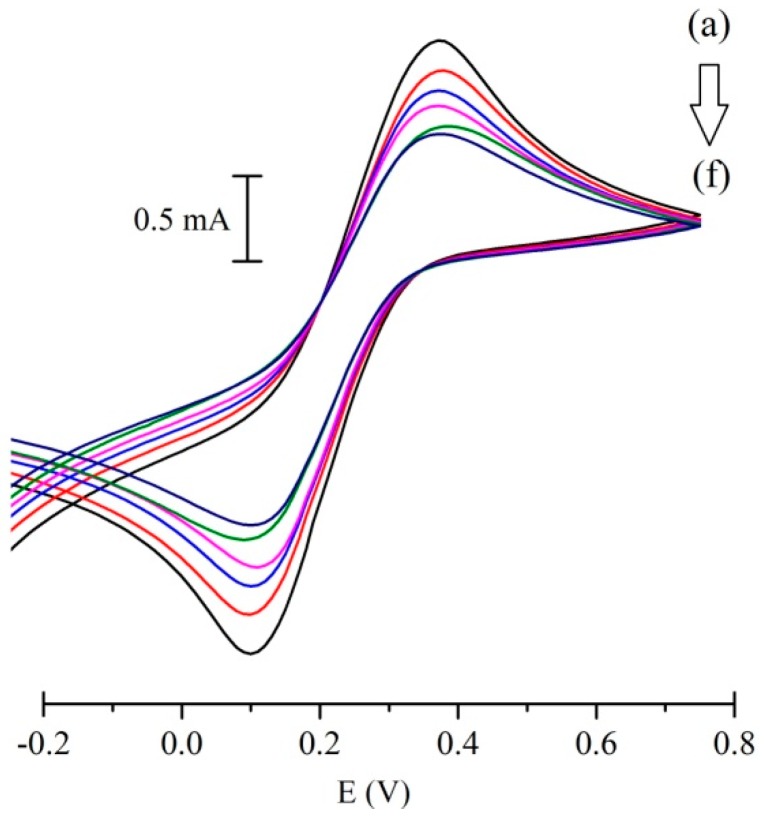
MIP-CP electrode responses to histamine. Cyclic voltammograms in different molar concentrations of histamine: 1 × 10^−10^ (**a**); 2 × 10^−9^ (**b**); 4 × 10^−9^ (**c**); 7 × 10^−9^ (**d**); 2 × 10^−7^ (**e**); and 4 × 10^−7^ (**f**). All of the histamine solutions were made in 0.1 M solution of hexacyanoferrate (III) and KCl as blank.

**Figure 3:**
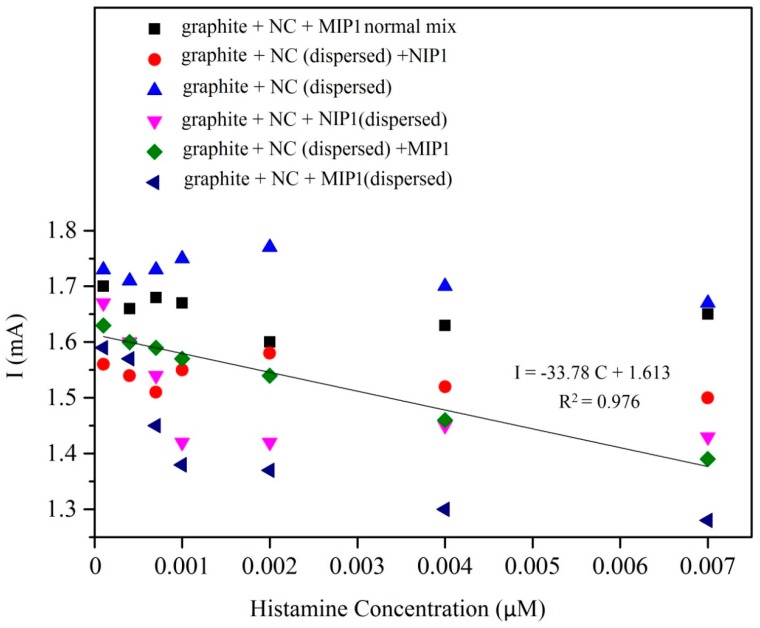
Comparison of voltammetric responses to histamine for different electrode composites. All of the histamine solutions were made in the 0.1 M solution of hexacyanoferrate (III) and KCl as a blank.

In the first steps of the fabrication process for optimization of composite, we have tested different sequences of mixing of nano carbon (NC), graphite and MIP/NIP as follows: (1) graphite + NC + MIP/NIP normal mix, these powders were physically mixed together as a dry powder; (2) graphite + NC + MIP/NIP (dispersed) powders were mixed together and dispersed in DMF, sonicated, and dried; (3) graphite + NC (dispersed) + MIP/NIP, graphite, and NC was dispersed in DMF, sonicated, and dried followed by mixing of MIP/NIP powder as described in material and methods. Paraffin oil was added to prepare the paste in all the composites. Subsequently, electrochemical responses of hexacyanoferrate solution with different concentrations of histamine were investigated for all electrodes (Figure 2). The MIP-CP electrode prepared by the graphite + NC (dispersed) + MIP/NIP method gave a linear electrochemical signal in cyclic voltammetric measurement (Figure 3). This is most likely related to accessibility of MIP for recognition of histamine in the composite materials.

#### 3.2.2. Selectivity of the Modified CP Electrode

**Figure 4:**
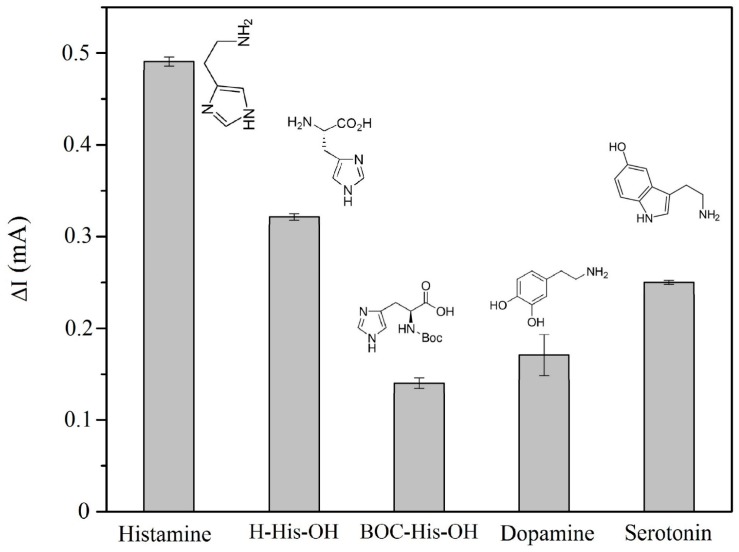
Selectivity investigation by cyclic voltammetry; electrochemical response of MIP-CP electrode for histamine and other similar structure compounds. [Histamine and all analytes] = 0.004 µM, scan rate = 50 mV/s, E_step_ = 10 mV and equilibration time = 5 s.

The MIP-CP electrode was evaluated using structurally-similar compounds and neurotransmitters. In order to accomplish these experiments, the MIP-CP electrode was immersed in individual solution (concentration = 7 × 10^−9^ M) and the electrochemical detection process was carried out according to the above mentioned procedure. Figure 4 illustrates the responses of histamine and other histamine-like compounds, including the neurotransmitters serotonin and dopamine, in the histamine MIP-CP electrode under the exact same conditions. As can be seen, the responses of histamine to the MIP-CP electrode is the highest compared to other analytes, followed by H-His-OH amino acid having a similar structure. It is interesting to note that when the MIP-CP electrode was tested for Boc-His-OH, a lower response was observed. This may suggest that primary amine of histamine and MAA is important for interaction involved in molecular recognition.

### 3.3. Analytical Characterization

#### 3.3.1. Calibration of the MIP-CP Electrode

**Figure 5:**
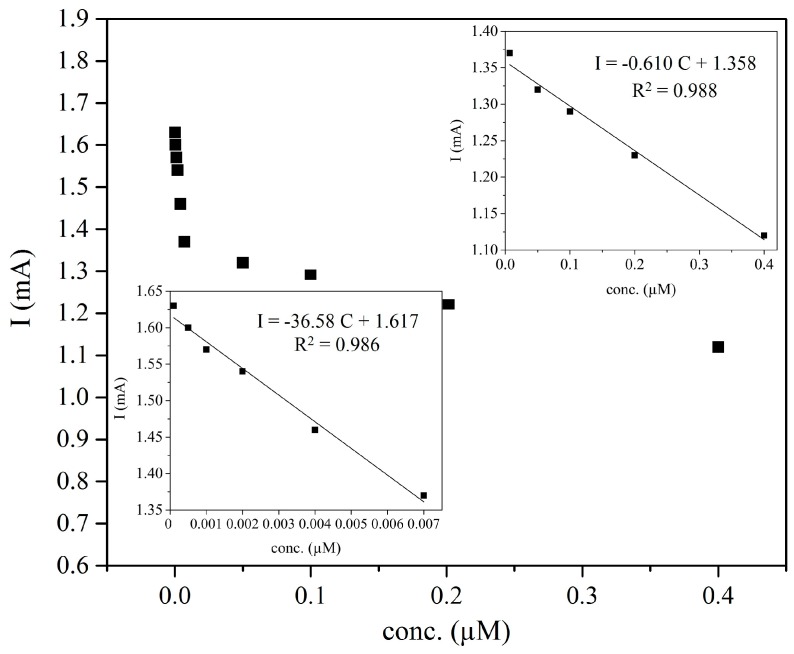
Calibration curve obtained for the developed sensor; (insets are showing two linear ranges of histamine, 1 × 10^−^^10^–7 × 10^−9^ M and 7 × 10^−9^–4 × 10^−7^ M).

The developed sensor was used for calibration curve plotting. It is worth noting that the values of the current response used for the calibration curve are actually the absolute values of the oxidative peak current observed for blank solution and after spiking of different concentrations of histamine solutions. The calibration graph obtained for histamine determination of the prepared sensor is shown in Figure 5 that exhibited two distinct linear response ranges of 1 × 10^−10^–7 × 10^−9^ and 7 × 10^−9^–4 × 10^−7^ M with the detection limit of 7.4 × 10^−11^ M (S/N = 3).

#### 3.3.2. Histamine Determination in Human Plasma

The analytical utility of the method was assessed by applying it to the determination of histamine in human serum samples. Percent recovery of histamine was obtained demonstrating that the proposed sensor is a promising approach in sensor preparation and histamine analysis. The results for the determination of histamine in different samples are summarized in Table 2.

### 3.4. Comparison of the Developed Method and Other Previously Reported Electrochemical Methods

In Table 3, the analytical parameter of reported electrochemical methods for quantification of histamine are summarized. Compared to most of other methods, the approach presented here shows a wide dynamic range and has a lower detection limit. The proposed procedures for making MIP-CP electrode and analytical method developed using bulk imprinted polymer is very simple and inexpensive. In this proposed sensor, the low detection limit, wide linear working range, and comparable sensitivity and selectivity to the advanced instrumentation is noteworthy.

## 4. Conclusions

Histamine, a biogenic amine, is an indicator in pathophysiology, microbial infection and food safety. Routinely, faster detection of histamine is performed by time-consuming chemical methods or ELISA. Therefore, MIPs, as synthetic receptors, are a cost-effective alternative. In this study, histamine-imprinted polymers were prepared by bulk imprinted polymerization in chloroform and acetonitrile. The imprinted polymer prepared in chloroform displayed better imprinting performance in a neutral buffer. Modification of carbon paste (CP) electrode with histamine MIPs as recognition elements lead to an excellent sensor for histamine. As a proof of principle, the measurement of histamine in the human serum is demonstrated using MIP-based voltammetric sensor.

## Figures and Tables

**Table 1:** Composition of histamine imprinted polymer by bulk polymerization.

Polymer	Template (mmol)	MAA (mmol)	EGDMA (mmol)	Solvent
MIP1	0.24	1.2	6	CHCl_3_
NIP1	-	1.2	6	CHCl_3_
MIP2	0.24	1.2	6	MeCN
NIP2	-	1.2	6	MeCN

**Table 2:** Determination of histamine in human serum.

Sample	Spiked (mol·L^−1^)	Found (mol·L^−1^)	Recovery (%)	RSD (%)
Human serum	5.0 × 10^−10^	5.2 × 10^−10^	104	2.02
	4.0 × 10^−9^	4.2 × 10^−9^	105	3.58
	2.0 × 10^−7^	1.9 × 10^−7^	95	3.42

**Table 3:** Comparison of analytical parameters of the proposed sensor and some other previously-reported histamine electrochemical sensors.

Method	Electrode	Linear Range (mol·L^−1^)	Detection Limit (mol·L^−1^)	Reference
Impedimetry	Polymer-coated Al	1.2 × 10^−^^8^–2.0 × 10^−^^9^	2.0 × 10^−^^9^	[42]
Voltammetry	Glassy carbon	2.0 × 10^−^^4^–5.0 × 10^−^^6^	0.3 × 10^−^^6^	[21]
Amperometry	Screen-printed	6.0 × 10^−^^5^–8.0 × 10^−^^6^	8.1 × 10^−^^6^	[45]
Amperometry	Heterogeneous carbon	8.9 × 10^−^^5^–4.5 × 10^−^^6^	1.8 × 10^−^^6^	[46]
Voltammetry	SWCNT-modified carbon paste	7.2 × 10^−^^4^–4.5 × 10^−^^6^	1.3 × 10^−^^6^	[20]
Chronopotentiometry	Gold	8.9 × 10^−^^4^–1.8 × 10^−^^5^	2.4 × 10^−^^6^	[28]
Chronopotentiometry	Glassy carbon	8.1 × 10^−^^4^–1.8 × 10^−^^5^	1.2 × 10^−^^5^	[47]
Amperometry	Boron-doped diamond	8.1 × 10^−^^3^–4.5 × 10^−^^5^	4.0 × 10^−^^5^	[48]
Voltammetry	Gold micro electrode	4.9 × 10^−^^8^–9.9 × 10^−^^12^	3.1 × 10^−^^12^	[49]
Voltammetry	NC/MIP/CPE	4 × 10^−7^–7 × 10^−9^ and 7 × 10^−9^–10 × 10^−^^10^	7.4 × 10^−^^11^	This work
